# Supplementary material for: Are carbon emissions trading and green financial instruments synergistic? -Comprehensive quantitative research based on content analysis
Source: PLoS One. 2024 Mar 7;19(3):e0298601. doi: 10.1371/journal.pone.0298601 (PMC10919601; doi:10.1371/journal.pone.0298601)
Supplement: S1 Data — (ZIP) [file pone.0298601.s001.zip › Data/地级市Z_F工作报告.zip!/┴╔─■/┴╔╤⌠╩╨/└┤╫╘╣1⁄2╓┌║┼í╛┬φ┐╦╩2╛▌═°í┐_2015.docx]

各位代表：

我代表市人民政府向大会报告工作，请予审议，并请市政协委员和列席会议的同志提出意见。

一、2014年工作回顾

刚刚过去的2014年，是经济下行压力加大，发展较为困难的一年，也是我们积极应对挑战，保持经济平稳增长的一年。一年来，在省委、省政府和市委的正确领导下，市政府团结带领全市人民，认真贯彻党的十八大和十八届三中、四中全会精神，坚持稳中求进的工作总基调，砥砺奋进，攻坚克难，促进了经济平稳发展、民生持续改善、社会和谐稳定。

预计，全年地区生产总值增长6%；一般公共预算收入增长3.1%；固定资产投资增长6%；城乡常住居民人均可支配收入分别增长10%和11%。

(一)以河东为龙头的新城建设加快推进

河东新城建设取得新进展。基础设施日趋完善，襄平大桥合龙，新运大桥西侧道路取直通车。高标准打造太子河历史文化风光带，“一河两岸”环境明显提升，成为市民休闲健身的理想场所。进一步理顺新城管理体制，管理水平不断提高。一批便民服务业项目相继入驻。培育新城主导产业取得重大进展，忠旺项目落户铝合金精深加工园区，产业大道基本建成。大型专业市场建设步伐加快，红美五金建材交易中心投入运营，叠石桥国际家纺城与中南新城购物中心正式签约。各类商住项目加快建设。融资工作实现突破，28亿元私募资金正式发行。

首山、灯塔、汤河、佟二堡4个新城建设稳步推进，基础设施不断完善，区域环境明显优化，承载能力显著增强。

(二)工业经济稳步增长

加强工业经济运行监测调度，落实包扶制度，帮助企业解决困难和问题，与企业共度难关。出台扶持政策，落实财政补贴，减轻企业负担，提振发展信心，促进工业经济平稳增长。

工业结构调整取得积极进展。俄油加工优化增效改造项目前期工作进展顺利。200万吨铝板带箔项目全面开工建设，8万辆铝合金专用车厢项目加快实施，工业铝材产业优势日益凸显。芳烃基地连续两年入围全国化工园区20强，23万吨苯深加工、天然气制氢项目即将投产。经济开发区承载力、吸引力显著提升，500万支法兰轴头及轮毂项目一期建成投产。钢铁产业结构进一步优化，累计淘汰小钢铁企业91家，3个钢铁转型升级项目进展顺利。

科技创新能力明显增强。与中科院、大连理工、东北大学等院校建立了紧密合作关系，产学研合作水平进一步提升。中科院沈阳国家技术转移中心落户高新区。三三工业公司完成了海外并购和技术引进，核心技术和生产能力达到世界先进水平。全年新增企业技术中心、公共技术服务平台19个。软件信息等新兴产业加快发展。

环境保护力度加大。青山工程向纵深发展，实施经济林工程建设3.9万亩，闭坑矿山生态治理年度任务全面完成。蓝天工程强力推进，加速淘汰落后产能，突出抓好重点行业脱硫、脱硝和油气回收治理，城市空气环境质量得到改善。碧水工程进展顺利，辽河流域水污染治理不断加强。万元地区生产总值综合能耗下降4%，主要污染物实现达标排放。

(三)农业农村工作开创新局面

现代农业建设步伐加快。我市获批国家现代农业示范区，小小线两侧重点区域被确定为省级现代农业示范区。建成现代化农业生产示范区32万亩，创建国家级粮食高产示范区13个。积极抗旱减灾，粮食总产量实现95万吨。强化农产品质量安全监管，全年无农产品质量安全事故发生。农业产业化加快发展，开工建设投资亿元以上项目6个，辽阳县荣获“中国平欧大果榛子之乡”称号，佟二堡被命名为“中国皮草之都”。鲜活农产品流通体系初步形成，认定农产品供应基地5万亩，建成中心城区农产品直销卖场1处、直销点21个。

新型农村经营体系初步建立。基本完成农村土地承包经营权确权登记颁证试点工作，流转土地60万亩，新增农民专业合作社290家、家庭农场384家。创新农村金融服务体系，完善农业担保平台，缓解了农业生产融资难问题。积极开展农业保险和核灾理赔工作。

农业农村基础设施不断完善。改造农田基础设施32万亩，实施水利骨干工程71项。新建农村安全水源54处，解决了13.2万人的饮水安全问题。对188个行政村实施了综合整治，亮化村庄491个，绿化村屯60个，建成村内道路291公里，建设美丽乡村示范村15个。

山区扶贫开发取得新进展。完成了贫困人口建档立卡工作，建立扶贫资金互助社10个。年纯收入3200元以下的农村贫困人口中，有1.8万人实现稳定脱贫。

(四)服务业发展迈上新台阶

制定《服务业发展四年行动计划》，明确任务措施，设立引导资金，充分激发了服务业发展的内生动力。

服务业集聚区建设成效显著。辽阳友谊商城正式营业，兴隆大家庭开工建设，中南望京购物中心进展顺利，提升了白塔中央商务区的影响力。站西商务区启动建设。河东新城跻身省级现代服务业集聚区行列。灯辽高速竣工通车，海宁皮革城三期投入运营，佟二堡保税仓库基本建成，增强了皮装裘皮商贸区的集聚能力。西土线改扩建工程大部分完工，水天一色温泉度假酒店、巴塞罗那国际温泉娱乐中心等项目加快推进，温泉旅游度假区服务功能日益完善。

生产性服务业加速发展。金融业发展步入快车道，锦州银行、恒安人寿等金融企业相继入驻我市。金融业税收大幅增长，占第三产业税收比重达到20%。物流业发展迈出新步伐，第地嘉公司晋升为全省现代物流示范企业。全面完成了39户企业分离生产性服务业工作。传统商业加快向电子商务转变，辽阳易购网上商城正式营业，“指掌四海”等电子商务平台建设步伐加快。

旅游业蓬勃发展。“温泉冰雪、皮装裘皮购物游”两大特色品牌持续升温，龙石风景区荣获国家4A级旅游景区称号。

消费市场日趋活跃。成功举办了汽车、台湾名品、地产名优商品等各类展销会，夜经济逐渐成为新的消费热点。

(五)城市建设和管理水平不断提高

房地产业健康平稳发展。抓住国家加快棚户区改造的有利契机，启动了大规模的改造工程，实施棚改项目34个，开工建设房屋3万套，涉及居民3万多户。狠抓房地产销售，成功举办春秋两季房交会。全年销售房地产193万平方米，实现销售额82亿元。

人居环境不断优化。护城河景观带建设加快推进，关帝园和喇嘛园两处游园建成并投入使用，成为广受市民欢迎的休闲健身场所。改造振兴路、繁荣路两侧绿化带，提升了城市出入口形象。综合改造老旧小区21个，维修“弃管楼”242栋，对61栋老旧楼房进行了保温处理，市民生活环境和居住条件明显改善。

城乡基础设施进一步完善。维修改造城市支路和街巷道路20条，建成襄平贸易城过街天桥，购置121台新能源公交车并投入运营，方便了群众出行。市净水厂、河东净水厂通水运行，改造城市供水设施45处，基本完成市政供水区域内封闭地下井工作。新建改造燃气管网10.6公里，中心城区燃气普及率达到95%。备用热源二期工程投入使用。大中修和改扩建县级以上公路285公里、农村公路577公里，建设里程和资金投入再创新高。

城市管理得到加强。深入开展侵占居民小区公共场地整治行动，清理占道经商，取缔马路市场，规范破路施工行为，强化环境卫生治理，市容市貌有新改观。

(六)改革开放进一步深化

全面贯彻落实国务院28号文件精神，明确130项任务分工，确定了争取政策的时间表和路线图，一批重大项目前期准备工作全面展开。

围绕转变职能和理顺职责关系，积极推进新一轮机构改革，政府工作部门整合为32个，组织体系更加精干、高效。大力简政放权，取消行政职权113项，下放82项。市属国有企业改革不断深化，4户企业完成国有股退出。民营企业体制机制创新步伐加快，科隆精化成功上市，全市上市民企已达3家。工商登记制度改革激发了创业投资热情，新增公司制企业1430户。医药卫生体制改革稳步推进，回收承包乡镇卫生院17家；新城、襄平、太子河医院成功引进社会资本，多元化办医格局初步形成。

进一步扩大对外开放，瞄准长三角、珠三角等发达地区，坚持走出去、请进来，开展招商活动，促成一批项目签约落地。各项出口扶持政策全面落实，5个出口基地建设加快推进。实际利用外资增长15.4%。外贸出口增长2.4%。

(七)以保障和改善民生为重点的社会建设全面加强

年初确定的24项民生工程全面完成，有效解决了群众关心的衣食住行、就医就学、休闲健身、社会治理等一系列问题，广大市民的幸福感明显增强。

就业社保工作成效显著。大力开展就业援助，新增城镇实名制就业4.5万人，城镇登记失业率控制在2.5%以内，零就业家庭保持动态为零。完善社会保障体系，新增五险扩面6.1万人，解决了4.4万名破产和无缴费能力企业退休人员的医疗保障问题，合并实施城乡居民养老保险，启动了城乡居民大病保险。进一步提高保障标准，退休人员养老金月均提高171元，城乡居民基础养老金月均提高15元。新建廉租房600套，解决了城市部分低收入家庭的居住问题。积极推进社会养老服务体系建设，我市入选全国首批养老服务业综合改革试点地区。

各项社会事业全面进步。各级各类教育协调发展。城区教育质量进一步提高，基本实现义务教育均衡发展。完成了56所学校标准化建设，改善了49所学校的校园环境，新改扩建公办幼儿园6所，市直公立幼儿园全面竣工。强化校园卫生防疫与食品安全管理，校车安全得到保障。公共卫生服务全面加强，实施了二院、三院、九院病房楼改扩建工程，市中心医院在全省三甲医院运行与管理评价中名列前茅。免费开放市县乡村四级文化场所，建设社区书屋28个。民俗博物馆和24小时自助图书馆正式对外开放。雷锋纪念馆被命名为全国爱国主义教育示范基地。建成社会体育指导站200个，就近指导了广大群众科学健身。参加省十二运取得历史最好成绩。残疾人事业加快发展，康复中心建成并投入使用。深入开展关爱职工送温暖活动，解决了部分困难职工的实际问题。

国防教育和国防动员建设进一步加强，创建全国文明城市步伐加快。国家安全、民族宗教、外事侨务、涉台事务、海关、检验检疫、防震减灾、人防、人口和计划生育、物价、地方志、住房公积金、统计、档案、慈善、红十字和妇女儿童等事业都取得了新的进步。

社会管理综合治理取得新成效。坚持打击整治“两个轮子”一起转，大力推进警力下沉，健全立体化社会治安防控体系，人民群众安全感明显增强。集中化解信访积案，维护了社会和谐稳定。落实安全生产“党政同责、一岗双责”规定，安全生产形势保持平稳。质量强市工作全面启动。建立食品药品监管新机制，保障了群众饮食用药安全。建成市突发事件预警信息发布平台。创新社区治理，白塔区再次荣获“全国和谐社区建设示范区”称号。

(八)民主法制建设扎实推进

主动接受市人大及其常委会的法律监督、工作监督和市政协的民主监督，认真执行市人大及其常委会的决议和决定，认真听取各民主党派、工商联、无党派人士、人民团体的意见和建议，支持各人民团体依照各自章程开展工作。全年办理人大代表议案、建议193件，政协提案308件，办复率99%、满意率100%。

认真贯彻落实党的十八届四中全会精神，加快推进法治政府建设，依法行政水平进一步提高。“六五”普法深入开展，法律服务和法律援助不断加强。落实党风廉政建设责任制，以“五大系统”为重点的惩防体系建设成效明显，公共资源交易系统建设取得重大进展，行政监察和审计监督得到强化。

扎实开展党的群众路线教育实践活动，狠刹“四风”，切实整改，公务接待进一步规范，机关服务意识和行政效率全面提高，政风行风建设显著加强。市县乡村四级公共服务体系初步建立，联系服务群众的渠道更加畅通。

各位代表，在去年经济形势严峻复杂的局面下，取得上述成绩极为不易。这些成绩的取得，是全市人民团结奋斗的结果；是市人大法律监督、工作监督和市政协民主监督、参政议政的结果；是驻军部队，中央、省驻我市单位，各民主党派、人民团体、社会各界人士、外来投资者和建设者关心支持的结果。在此，我代表市政府，向支持参与辽阳经济社会建设的全市人民和各界人士表示衷心的感谢！

在总结成绩的同时，我们也清醒地看到经济社会发展中，还存在许多矛盾和问题。主要是：需求总体偏弱，经济下行压力加大；结构调整任务艰巨，新的经济增长点尚未形成，产业转型升级任重道远；城市建设管理任务繁重，新型城镇化质量不高；财政收支矛盾仍然突出，收入质量有待进一步提高，保障和改善民生的任务还很艰巨；政府职能转变还不到位，“四风”问题不同程度地存在。对于这些问题，我们将采取有效措施，切实加以解决。
